# Volatile concentrate from the neotropical moss *Neckeropsis undulata* (Hedw.) Reichardt, existing in the brazilian Amazon

**DOI:** 10.1186/s13065-021-00736-3

**Published:** 2021-01-25

**Authors:** Thyago G. Miranda, Raynon Joel M. Alves, Ronilson F. de Souza, José Guilherme S. Maia, Pablo Luis B. Figueiredo, Ana Cláudia C. Tavares-Martins

**Affiliations:** 1 Programa de Pós-Graduação em Biodiversidade e Biotecnologia, Universidade Federal do Pará, Belém, PA 66075-110 Brazil; 2 Departamento de Ciências Naturais, Universidade do Estado do Pará, Belém, PA 66050-540 Brazil; 3 Programa de Pós-Graduação em Química, Universidade Federal do Maranhão, São Luís, MA 64080-040 Brazil

**Keywords:** Neckeraceae, Volatile concentrate, 1-octen-3-ol, α-muurolol, *n*-hexanal

## Abstract

**Background:**

Many natural compounds have been identified and synthesized by the advancement of bryophytes phytochemistry studies. This work aimed to report the composition of *Neckeropsis undulata* (Hedw.) Reichardt moss volatiles, sampled in the Combú Island, Belém city, Pará state, Brazil. The volatile concentrate of *N. undulata* was obtained by a simultaneous distillation-extraction micro-system, analyzed by GC and GC-MS, and reported for the first time.

**Results:**

Ten compounds were identified in the volatile concentrate, corresponding to 91.6% of the total, being 1-octen-3-ol (35.7%), α-muurolol (21.4%), naphthalene (11.3%), and *n*-hexanal (10.0 %) the main constituents. Most of the constituents of the *N. undulata* volatile concentrate have been previously identified in other mosses, and liverworts spread wide in the world.

**Conclusions:**

1-Octen-3-ol, *n*-hexanal, 2-ethylhexanol, isoamyl propionate, and octan-3-one are already known metabolic products obtained from enzymatic oxidation of polyunsaturated fatty acids, belonging to the large family of minor oxygenated compounds known as oxylipins. The knowledge of the composition of volatiles from moss *N. undulata* could contribute to the Neckeraceae species’ chemotaxonomy.

## Background

Bryophytes are small terrestrial spore-forming green plants, phylogenetically placed between vascular plants and algae, and comprises about 22,000 species worldwide. They are most diverse in the tropics and fit in a general pattern of various groups of organisms, increasing species toward the equator line. Mosses (Bryophyta), liverworts (Marchantiophyta), and hornworts (Anthocerotophyta) compose the three lineages of bryophytes. The mosses are estimated to include about 12,700 species [1]. Brazil’s bryophyte flora comprises 1524 species, of which 880 are mosses, 633 liverworts, and 11 hornworts. The Atlantic Forest biome has the most significant number of species (1337), followed by the Amazon (570) and the Cerrado (478) biomes [2].

Neckeraceae, a moss family with about 200 species, belongs to the order Hypnales of pleurocarpous mosses. *Neckeropsis undulata* (Hedw.) Reichardt [syn. *Daltonia undulata* (Hedw.) Arn., *Distichia undulata* (Hedw.) Brid., *Eleutera jamaicensis* Stuntz, *Fontinalis crispa* Sw., *Neckera amazonica* Mitt., *N. undulata* Hedw., *Pilotrichum undulatum* (Hedw.) P. Beauv.], a neotropical moss species widespread in all Americas, is light to a dark green plant, stems 5.0 mm long and perpendicular to the substrate, leaves ovate-ligulate 2.0 mm long to 1.0 mm wide, forming mats, frequently occurring on trunks of shrubs and trees [3–5].

Bryophytes have no physical defenses against any type of threat and, in general, are not attacked by insects and small animals. These plants emit scents such as sweet-woody, sweet-mossy, turpentine, carrot-like, mushroomy, and seaweed-like. However, bryophytes’ volatiles chemistry is not well known because they are small species and challenging to collect. Besides, they are considered of little nutritional value to humans. However, some mosses have been widely used as medicinal plants in China to cure burns, bruises, external wounds, snake bite, fractures, and convulsions [6, 7].

The essential oil composition of some moss species has been previously reported. In the oils of *Hylocomium splendens* (Hedw.) Schimp. and *Leucodon sciuroides* (Hedw.) Schwagr., existing in Turkey, the primary constituents were β-pinene and α-pinene, and nonanal and heptanal, respectively [8]. The presence of sesquiterpene and diterpene hydrocarbons in the oils of six mosses species from Ecuador were reported: in *Breutelia tomentosa* (Sw. ex. Brid.) A. Jaeger the primary constituents were epizonarene and α-selinene; in *Leptodontium viticulosoides* (P. Beauv.) Wijk & Margad. were α- and β-selinene; in *Macromitium perreflexum* Steere were selina-3,11-dien-6-α-ol and curcuphenol; in *Campylopus richardii* Brid. were *epi*-α-muurolol and α-cadinol; in *Rhacocarpus purpurascens* (Brid.) Paris. were α-cadinol and α-santalene, and in *Thuidium peruvianum* Mitt. were phytol and valerenol [9]. Phytol and 1-octen-3-ol were the primary components obtained in the oil of the moss *Rhodobryum ontariense* Kindb. From Serbia [10]. Linoleic acid, arachidonic acid, and eicosapentaenoic acid are common in bryophytes, and they act as biosynthesis precursors of some mushrooms smelling eight-carbon alcohols and ketones, metabolites known as oxylipins, such as 1-octen-3-ol and octan-3-one [11–13].

Essential oils analysis of *Neckera complanata* (Hedw.) Huebener and *N. crispa* Hedw., species also occurring in Turkey and belonging to the Neckeraceae have been described. β-Phellandrene, camphene, γ-bisabolene, and α-pinene were the main compounds in the *N. complanata* oil, while octan-3-one and limonene predominated in the oil of *N. crispa* [14].

Few chemical and biological studies have been done with bryophytes in Brazil. Organic extracts of 25 bryophytes from the Brazilian Amazon, with solvents of different polarities, were tested against 13 pathogenic bacteria of a broad general spectrum [15]. The antibacterial activity of ethanol extract of the moss *Octoblepharum albidum* from Crato, Ceará state, Brazil, alone and in association with aminoglycosides, was determined against bacterial strains, resulting in significant and similar inhibitory activity for *Escherichia coli* ATCC 25,922 and *Klebsiella pneumoniae* ATCC 33,018. Also, phytochemical screening was performed with the same extract to identified the bioactive compounds [16]. Chemical diversity of the secondary metabolites of 12 populations of *Syzygiella rubricaulis* (Nees) Stephani (Jamesoniellaceae, Marchantiophyta) from the high tropical mountains of Mexico, Venezuela, Ecuador, and Brazil was performed. Sesquiterpenoids, diterpenoids, and long-chain hydrocarbons predominated in the CHCl_2_ extracts of the 12 populations of *S. rubricaulis* [17]. To the best of our knowledge, no work with essential oils or volatile concentrates of bryophytes has been previously published in Brazil.

This work aimed to report the volatile composition from the moss *Neckeropsis undulata* (Hedw.) Reichardt, which occurs in the Brazilian Amazon, in order to extend the chemotaxonomic knowledge of this species.

## Experimental

### Plant material and extraction procedure

*Neckeropsis undulata* (Hedw.) Reichardt (Neckeraceae) is a native and non-endemic moss species, with occurrence throughout all Brazil regions [5] and easily found in the Brazilian Amazon. The samples of *N. undulata* were collected in a floodplain area close to Belém, Pará state, Brazil, on the trunk of live cocoa trees, using techniques proposed by Yano (1984) [18]. Different bryophytes can occur together, and this species was separated using tweezers with magnifying glasses, considering its gametophyte and sporophyte phases. The botanical identification was carried out by Prof. Ana Cláudia Martins, a Bryophytes specialist from Departamento de Ciências Naturais, Universidade do Estado do Pará, Belém, Pará state, Brazil. The moss sample (100 g) was air-dried for three days, and its volatile concentrate was obtained by micro hydrodistillation-extraction in a Likens-Nickerson apparatus (25 g, 2 h, duplicate) [19], using n-pentane (3 mL) as the solvent.

### Volatile concentrate analysis

The volatile concentrate of *N. undulata* was submitted to GC and GC-MS analysis. It was performed on a GCMS-QP2010 Ultra system (Shimadzu Corporation, Tokyo, Japan), equipped with an AOC-20 i auto-injector and the GCMS-Solution software containing the NIST and FFNSC 2 libraries [20, 21]. A Rxi-5 ms (30 m × 0.25 mm; 0.25 µm film thickness) silica capillary column (Restek Corporation, Bellefonte, PA, USA) was used. The conditions of analysis were as follows. Injector temperature: 250 °C; Oven temperature programming: 60–240 °C (3 °C/min); Helium as the carrier gas, adjusted to a linear velocity of 36.5 cm/s (1.0 mL/min); split mode injection (split ratio 1:20) of 1.0 µL of the *n*-pentane solution; ionization by electronic impact at 70 eV; ionization source and transfer line temperatures of 200 and 250 °C, respectively. The mass spectra were obtained by automatic scanning every 0.3 s, with mass fragments in the range of 35–400 m/z. The retention index was calculated for all volatile components using a homologous series of C8-C40 *n*-alkanes (Sigma-Aldrich, Milwaukee, WI, USA), according to the linear equation of Van Den Dool and Kratz [22]. Individual components were identified by comparing their retention indices and mass spectra (molecular mass and fragmentation pattern) with those existing in the GCMS-Solution system libraries [20, 21, 23]. The quantitative data regarding the volatile constituents were obtained using a GC 2010 Series, operated under similar conditions to those of the GC-MS system. The relative amounts of individual components were calculated by peak-area normalization using a flame ionization detector (GC-FID). The analyses were performed in duplicate.

## Results and discussion

The yield of the volatile concentrate of the *N. undulata* moss was 0.25 %. Thirteen constituents are listed in Table 1, comprising 99.8 % of the total concentrate. 1-Octen-3-ol (35.7%), α-muurolol (21.4%), naphthalene (11.3 %), and hexanal (10.0 %) were the primary components.

The addition of molecular oxygen to polyunsaturated fatty acids and the transformations of the resulting hydroperoxides leads to the production of a large family of minor oxygenated compounds known as oxylipins. The main constituent of the volatile concentrate on *N. undulata* was 1-octen-3-ol (35.7%). It is allyl alcohol known as mushroom alcohol, and an oxylipin produced by enzymatic oxidation and cleavage of the linoleic, arachidonic, and eicosapentaenoic acids. In nature, the 1-octen-3-ol is indicated as a signaling molecule in plant cellular responses, plant-herbivore interactions, and plant-plant interactions [11, 13, 24]. In the oil of the moss *Rhodobryum ontariense* Kindb. from Serbia, 1-octen-3-ol has been identified as the secondary constituent, following the diterpene phytol, which was the main component [10]. The thalli of the liverwort *Marchantia polimorpha* emitted high amounts of C8 volatiles, after mechanical wounding, mainly consisting of 1-octen-3-ol and octan-3-one, which were also isolated from the *N. undulata* volatile concentrate, together with another unidentified C8 alcohol, with a low percentage. The presence of arachidonic acid or eicosapentaenoic acid seemed essential for the formation of these volatile compounds of C8-type [25].

**Table 1:** Constituents identified in the volatile concentrate of *Neckeropsis undulata*

Constituents (%)	RI_C_	RI_L_	Volatile concentrate (%)*
*n-hexanal*	*798*	*801*^**a**^	*10.0*
isoamyl propionate	969	960^a^	1.2
*1-octen-3-ol*	*974*	*974*^**a**^	*35.7*
unidentified 1-octen-3-ol isomer, MW 128	979		0.6
octan-3-one	983	979^a^	1.0
2-pentylfuran	989	984^a^	1.6
2-ethylhexanol	1025	1030^b^	2.8
*naphthalene*	*1180*	*1178*^**a**^	*11.3*
benzophenone	1624	1626^a^	1.7
*α-muurolol (= torreyol)*	*1645*	1644^a^	*21.4*
phytone	1842	1841^b^	4.9
unidentified oxygenated sesquiterpene, MW 222	1865		2.8
unidentified oxygenated sesquiterpene, MW 222	1959		4.8
Total (%)			99.8

RI_C_: Calculated Retention Index (Rxi-5 ms column); RI_L_: Literature Retention index

^a^ Adams [23]; ^b^ Mondello [21]; Main constituent in italics

* Constituent amounts were calculated by peak-area normalization using GC-FID; n = 2 (standard deviation was less than 0.1)

The second most abundant constituent of the volatile concentrate on *N. undulata* was the oxygenated sesquiterpene α-muurolol (21.4 %). Terpene compounds are present in little amounts in mosses when compared to liverworts [26]. α-Muurolol has a cadinane skeleton, and it is found in liverworts and mosses. Many sesquiterpene and diterpene compounds are also identified in higher plants [6]. For example, in the liverwort *Frullania brasiliensis* Raddi and moss *Campylopus richardii* Brid. oils, the stereoisomer *epi*-α-muurolol was the primary constituent, with 32.18 % and 15.1 %, respectively [9, 27].

The third-highest constituent in the volatile concentrate on *N. undulata* was naphthalene (11.3 %). The hydrocarbon naphthalene has been identified in the liverworts *Plagiochila subdura* Inoue and *Triandrophyllum subtrifidum* (Hook. & Taylor) Fulford & Hatcher [28]. The aromatic derivatives, 3,4-dimethoxy-1,2-methylenedioxynaphthalene, 1,4-dimethoxy-1,2-methylenedioxynaphthalene, and 2,4,7-trimethoxynaphthalene were isolated from extracts of *Wettsteinia inversa* (Sande-Lacoste) Schiffner (syn. *Plagiochila inversa* Sande-Lacoste) and *W. schusterana* Grolle, two liverworts existing in Taiwan and New Zealand, respectively [29, 30]. Also, 1,2,3-trimethoxynaphthalene and 1,2,4-trimethoxynaphthalene were isolated from extracts of the liverwort *Adelanthus decipiens* sampled in the British Isles and South America [31].

The fourth primary constituent identified in the volatile concentrate on *N. undulata* was *n*-hexanal (10.0%), which must have been generated from arachidonic acid or linolenic acid by the action of a lipoxygenase, as previously reported for the liverwort *Marchantia polymorpha* L, producing *n*-hexanal and (*Z*)-3-hexenal after wounded plant tissues [32]. The volatile fractions of two samples of the moss *Rhodobryum giganteum* (Schwaegr.) Par. from China also presented *n*-hexanal as its primary constituent, with amounts of 19.7 % (Kaiyuan city, Yunnan Province) and 8.6% (Lushui county, Yunnan Province), respectively [33].

The diterpene phytone (hexahydrofarnesyl acetone), identified in the volatile concentrate of *N. undulata* (4.9 %), is commonly found in variable amounts of mosses essential oils, such as *Tortula muralis* Hedw. (2.2%), *Homalothecium lutescens* (Hedw.) H. Rob. (2.0 %), *Hypnum cupressiforme* Hedw. (3.0%), and *Pohlia nutans* (Hedw.) Lindb. (3.7%) [34], while phytol (phytone precursor) may be found in meaningful amounts in the oils of the mosses *Rhodobryum ontariense* (31.95%) [10] and *Thuidium peruvianum* (21.7%) [9].

2-Ethylhexanol was identified in the volatile concentrate of *N. undulata* (2.8%) and also detected in the mosses *Oxystegus tenuirostris* (Hooler & Taylor) A. Smith (4.13%) and *Eurhynchium striatum* (Schreb. ex Hedw.) Schimp (5.85 %), with occurrence in Turkey [35], and *Rhodobryum giganteum* (1.7 %) existing in China [33]. It is assumed that 2-ethylhexanol was also generated from linoleic acid by the action of a lipoxygenase.

Benzophenone, present in small amounts in the volatile concentrate on *N. undulata* (1.7%), was also identified in the moss essential oil of *Leucodon sciuroides* (0.8%), growing in Turkey [8]. Moreover, 2-pentylfuran, another minor constituent on *N. undulata* (1.6%), was also found in the moss *Rhodobryum giganteum* (0.9%) [33].

## Conclusions

In this work, the volatile composition of *Neckeropsis undulata* moss was reported for the first time, and it is assumed that this knowledge can contribute to a better understanding of the Brazilian bryophytes. 1-Octen-3-ol, the primary constituent, is already a known metabolic product, obtained from enzymatic oxidation of polyunsaturated fatty acids. α-Muurolol is a oxygenated sesquiterpene, with a cadinane skeleton, found in other mosses and of chemotaxonomic importance. 1-Octen-3-ol, *n*-hexanal, 2-ethylhexanol, isoamyl propionate, and octan-3-one belong to the large family of minor oxygenated compounds oxylipins, generated from long-chain fatty acids.

## Data Availability

All data generated or analyzed during this study are included in this manuscript.

## Abbreviations

GC: Gas chromatography
GC-MS: Gas chromatography-mass spectrometry
GC-FID: Gas chromatography-flame ionization detector
